# Clinical characteristics of acute promyelocytic leukemia manifesting as early death

**DOI:** 10.3892/mco.2013.155

**Published:** 2013-07-23

**Authors:** BAI HE, SHAOYAN HU, GUOQIANG QIU, WEIYING GU

**Affiliations:** 1 Department of Hematology, The First People’s Hospital of Changzhou (Third Affiliated Hospital of Suzhou University), Changzhou, Jiangsu 213003, P.R. China; 2 Department of Hematology and Oncology, Children’s Hospital of Suzhou University, Suzhou, Jiangsu 215003, P.R. China

**Keywords:** acute promyelocytic leukemia, immunophenotype, early death

## Abstract

Acute promyelocytic leukemia (APL) is currently considered to be a highly curable disease. However, early death (ED) remains a major cause of treatment failure in APL. The purpose of this study was to retrospectively review the morphological, immunophenotypic and molecular characteristics of 26 patients with APL resulting in ED. It was observed that elevated white blood cell (WBC) counts, lower fibrinogen concentrations, morphological variant M3v, CD34^+^ and the short form (S- or bcr3 form) of the *PML-RARα* transcript were significantly associated with ED, mainly due to cerebral hemorrhage. Admission on weekends or holidays without immediate diagnosis or prompt administration of treatment for APL resulted in intracranial bleeding and was the major cause of ED. Therefore, it is recommended that APL and coagulopathy management treatments are promptly initiated only upon morphological and clinical suspicion of APL on admission, in order to reduce the risk of severe bleeding and lower the rate of ED.

## Introduction

Since 1985, the introduction of all-trans retinoic acid (ATRA) and arsenic trioxide has revolutionized the treatment of acute promyelocytic leukemia (APL). APL is now considered to be a highly curable disease, with a complete response (CR) rate of 90–95% and a 5-year disease-free survival (DFS) of 74% (1–3). However, early death (ED) is one of the major causes of treatment failure in APL and is frequently due to severe intracranial bleeding. ED rates are reported to be <10% in modern clinical trials involving ATRA (4,5), whereas, in a cohort of 70 APL patients who received induction therapy at Stanford Hospital, 19 and 26% succumbed to the disease within 7 and 30 days of admission, respectively (6). Analysis of the SEER database indicates that the 30-day mortality rate has remained static over the same period, despite a significant increase in the 3-year overall survival, suggesting a much higher incidence of ED, mostly due to hemorrhagic complications (7). In the present study, the morphological, immunophenotypic and molecular characteristics of patients who developed ED were retrospectively reviewed for further investigation of the causes of ED.

## Patients and methods

### Patients and treatment methods

A total of 128 consecutive patients with newly diagnosed APL who were hospitalized at The First People’s Hospital of Changzhou (Third Affiliated Hospital of Suzhou University) and The Children’s Hospital of Suzhou University between May, 2003 and December, 2012 were retrospectively investigated. Diagnosis was initially established according to morphology and subsequently confirmed in all the cases via karyotyping and RT-PCR identification of the specific *PML-RARα* fusion gene. In all the cases, records were available on complete blood counts, bone marrow promyelocyte counts, immunomarkers, karyotype, *PML-RARα* fusion gene type, coagulation laboratory parameters and cause of death. All the patients received ATRA at a dosage of 45 mg/m^2^/day with or without arsenic trioxide at 10 mg/day, with the exception of those with onset of intracranial hemorrhage. Daunorubicin (45 mg/m^2^ × 4 days) or Idarubicin (12 mg/m^2^/day × 4 days) and intensive supportive therapy, such as empirical antibiotic therapy, transfusion of condensed red blood cells, fresh plateletpheresis and/or plasma, were administered when the white blood cell (WBC) count increased to >30×10^9^/l. ED was defined as death within 15 days from diagnosis.

### Statistical analysis

The t-test and Wilcoxon-Mann-Whitney test were used for the comparison of parametric and non-parametric methods, respectively and the Chi-square test was used to assess the differences between categories. P<0.05 was considered to indicate a statistically significant difference.

## Results

The results demonstrated that out of a total 128 APL cases, 26 (20.3%) developed ED. Out of the 26 ED patients, 16 were male and 10 were female, which was of no statistical significance compared to the 102 non-ED patients (58 males and 44 females, P=0.667). The median age of the patients who suffered ED was 38.5 years (range, 4–83 years), compared to 31.12 years (range, 13–73 years) in non-ED patients (P=0.078). The WBC count at presentation was higher in ED patients (median, 19.3×10^9^/l) compared to non-ED patients (median, 5.23×10^9^/l, P=0.000). The average fibrinogen concentration at presentation was lower in ED patients compared with that in non-ED patients (1.14±0.19 vs. 1.24±0.18, P=0.009). However, the hemoglobin levels, platelet counts and bone marrow leukemic promyelocyte (BMLP) percentage at presentation in ED patients did not exhibit statistically significant differences compared to those in non-ED patients. Notably, of the 26 ED patients, 18 (69.23%) were morphologic M3v type, 18 (69.23%) had the bcr3 type of *PML/RARα* and 17 (65.38%) were CD34^+^, compared to 32 (31.37%, P=0.000), 28 (27.45%, P=0.000) and 34 (33.33%, P=0.003) of the 102 non-ED patients, respectively. However, there were no statistical differences in CD2^+^, CD15^+^ and HLA-DR^+^ between ED and non-ED patients (Table I). All 26 cases succumbed to the disease within 15 days of admission. Of these cases, an 83-year-old male patient died due to severe retinoic acid syndrome manifesting with high WBC of 87×10^9^/l, acute pulmonary edema and renal failure on the 9th day following ATRA induction therapy; and a 42-year-old female patient was confirmed as M3v on the 5th day post radical resection of mastocarcinoma and succumbed to severe pulmonary infection and acute respiratory failure 8 days following ARTA therapy. The remaining 24 patients succumbed to intracranial bleeding prior to or following diagnosis. With regards to the causes of intracranial bleeding, an 11-year-old boy was admitted to the hospital with the sudden onset of a coma and the brain CT scan demonstrated multiple intracranial leukemic infiltrations complicated by hemorrhaging. A further 6 cases with onset of cerebral hemorrhage were confirmed by CT scan, 3 Rh-negative cases developed cerebral bleeding due to platelet transfusion shortage and the remaining 14 cases were admitted to the hospital on the weekend and progressively developed intracranial hemorrhaging due to failure of immediate diagnosis and prompt platelet, plasma and ATRA therapy. Thus, it was observed that elevated WBC counts, lower fibrinogen concentration, morphologic M3v, CD34^+^ type and the short form (S-, bcr3) of *PML/RARα* transcript were significantly associated with ED, mainly due to cerebral hemorrhaging.

## Discussion

Severe hemorrhages in APL are commonly attributed to the frequent diffuse activation of coagulation, hyperfibrinolysis and non-specific proteolytic activity and have also been reported to occur prior to the diagnosis of APL and initiation of treatment (4). Our data demonstrated that, in a cohort of 128 APL cases, the ED rate was 20.31%, which was in accordance with the results of previous studies (6,7), although slightly higher compared to those reported by modern clinical trials (4), mainly due to weekend admission without immediate diagnosis and effective therapy for coagulopathy. Although a general consensus exists on the need to confirm the diagnosis of APL at the genetic level, current guideline recommendations state that the disease should be managed as a medical emergency only upon morphological and clinical suspicion, with immediate therapeutic actions aimed at counteracting the coagulopathy, including platelet/plasma and prompt ATRA administration (8). Furthermore, atypical morphology of M3v, a high expression of CD34^+^ and the S-form (bcr3) of the *PML*/*RARα* fusion gene were associated with ED in APL. Therefore, clinicians should remain alert for APL cases manifested with the above clinical characteristics to prevent ED. Empirical suspicion and prompt treatment as APL for those admitted on weekends with onset of severe bleeding may gain valuable time and avoid ED due to APL to a certain extent.

## Figures and Tables

**Table I:** Clinical characteristics at diagnosis between APLs with and without ED.

Clinical characteristics	APLs with ED (n=26)	APLs without ED (n=102)	P-value
Gender (male/female)	16/10	58/44	0.667
Age (years)	38.50 (4–83)	31.12 (13–73)	0.078
WBC count ×10^9^/l (range)	19.3 (2.50–95.20)	5.23 (0.30–32.12)	0.000
BPC ×10^9^/l (range)	23 (11.00–58.30)	27.34 (9.00–59.30)	0.183
Hemoglobin (g/l)	82.23±15.11	87.67±14.91	0.100
Fibrinogen (g/l)	1.14±0.19	1.24±0.18	0.009
BMLP (%)	70.41±10.88	74.09±11.83	0.152
M3/M3v type	8/18	70/32	0.000
CD15^+^/CD15^−^	10/16	46/56	0.543
HLA-DR^+^/HLA-DR^−^	15/11	47/55	0.290
CD56^+^/CD56^−^	8/18	22/80	0.323
CD2^+^/CD2^−^	16/10	45/57	0.112
BCR1/BCR3	8/18	74/28	0.000
CD34^+^/CD34^−^	17/9	34/68	0.003

Elevated WBC counts (P=0.000), lower fibrinogen concentration (P=0.009), morphologic M3v (P=0.000), CD34^+^ (P=0.003) and short form (S-, bcr3) of *PML/RARα* transcript (P=0.000) were significantly associated with ED. APL, acute promyelocytic leukemia; ED, early death; WBC, white blood cell; BPC, blood platelet count; BMLP, bone marrow leukemic promyelocyte; M3v, acutre promyelocytic leukemia variant type.
